# Role of Rho guanine nucleotide exchange factors in non-small cell lung cancer

**DOI:** 10.1080/21655979.2021.2006519

**Published:** 2021-12-02

**Authors:** Rui-Jie Zeng, Wei-Jie Xie, Chun-Wen Zheng, Wan-Xian Chen, Si-Meng Wang, Zheng Li, Chi-Bin Cheng, Hai-Ying Zou, Li-Yan Xu, En-Min Li

**Affiliations:** a Department of Biochemistry and Molecular Biology, Shantou University Medical College, Shantou China; b The Key Laboratory of Molecular Biology for High Cancer Incidence Coastal Chaoshan Area, Shantou University Medical College, Shantou China; c Department of Gastroenterology, Guangdong Provincial People’s Hospital, Guangdong Academy of Medical Sciences, Guangzhou China; d Institute of Oncologic Pathology, Shantou University Medical College, Shantou China

**Keywords:** Rho GEFs, ABR, PREX1, DOCK2, DOCK4, NSCLC

## Abstract

Conventionally, Rho guanine nucleotide exchange factors (GEFs) are known activators of Rho guanosine triphosphatases (GTPases) that promote tumorigenesis. However, the role of Rho GEFs in non-small cell lung cancer (NSCLC) remains largely unknown. Through the screening of 81 Rho GEFs for their expression profiles and correlations with survival, four of them were identified with strong significance for predicting the prognosis of NSCLC patients. The four Rho GEFs, namely ABR, PREX1, DOCK2 and DOCK4, were downregulated in NSCLC tissues compared to normal tissues. The downregulation of ABR, PREX1, DOCK2 and DOCK4, which can be attributfed to promoter methylation, is correlated with poor prognosis. The underexpression of the four key Rho GEFs might be related to the upregulation of MYC signaling and DNA repair pathways, leading to carcinogenesis and poor prognosis. Moreover, overexpression of ABR was shown to have a tumor-suppressive effect in PC9 and H1703 cells. In conclusion, the data reveal the unprecedented role of ABR as tumor suppressor in NSCLC. The previously unnoticed functions of Rho GEFs in NSCLC will inspire researchers to investigate the distinct roles of Rho GEFs in cancers, in order to provide critical strategies in clinical practice.

## 1. Introduction

Lung cancer is one of the most common malignancies and a leading cause of cancer deaths (>1 million annually) worldwide [1,2]. Despite therapeutic advances, the prognosis of lung cancer remains poor, and more than half of the patients diagnosed with lung cancer die within one year [3]. Non-small cell lung cancer (NSCLC) constitutes approximately 85% of all lung cancers, of which lung adenocarcinoma (LUAD) and lung squamous cell carcinoma (LUSC) are the most common subtypes [3,4]. A comprehensive understanding of genetic alterations and associated mechanisms in the development of NSCLC is required to more effectively predict the prognosis of patients and identify druggable targets in cancer therapeutics.

Rho guanosine triphosphatases (GTPases) are essential molecular switches involved in the regulation of numerous downstream pathways in various types of cancer [5,6]. The cycling between guanosine triphosphate (GTP)-bound (active) state and guanosine diphosphate (GDP)-bound (inactive) state contributes to the activation or inactivation of downstream effectors [7]. In the ‘on’ state (GTP-bound) of Rho GTPases, target proteins are recognized and subsequently a response is generated until the Rho GTPases are turned to ‘off’ state (GDP-bound) [5]. Ras-related C3 botulinum toxin substrate 1 (RAC1), Ras homolog family member A (RHOA) and cell division control protein 42 homolog (CDC42), which are the most important and extensively studied members of Rho GTPases, have been identified to regulate actin cytoskeleton reorganization, migration, and metastasis and promote the development of lung cancer [8–11].

Rho GTPases activity is principally regulated by three types of regulatory proteins, including guanine nucleotide exchange factors (GEFs) for activation, GTPase-activating proteins (GAPs) for inactivation and GDP-dissociation inhibitors (GDIs) for GDP-bound form stabilization [12]. GEFs catalyze GDP release and subsequently assist in its binding to GTP, converting inactive Rho GTPases to their active states for further functioning [13]. As activators of Rho GTPases, Rho GEFs have attracted the attention of researchers in recent years. Rho GEFs can be divided into two distinct families: the diffuse B-cell lymphoma (DBL) family and the dedicator of cytokinesis (DOCK) family [14]. There are 70 members of the DBL family GEFs and 11 members of the DOCK family GEFs [14,15]. The altered expression or mutation of Rho GEFs has been identified in human cancers [16]. However, compared to the roles of Rho GTPase family members, the roles of Rho GEF family members in NSCLC remain largely unclear. Consequently, exploring the altered expression and relevant mechanisms of Rho GEFs leading to NSCLC development is significant.

Herein, we hypothesize that Rho GEFs can exert functions in addition to Rho GTPase activation in NSCLC. The aim of our study was to analyze the expression of Rho GEFs and their functions in NSCLC. Certain Rho GEFs will be selected for their observably altered expression and significant predictive value for prognosis in NSCLC patients. Moreover, methylation and mutation profiles were analyzed to interpret the observed altered expression of the selected members. The potential mechanisms related to the selected Rho GTPases in NSCLC development were investigated. *In vitro* experiments were performed to confirm the role of the selected Rho GTPase in NSCLC.

## 2. Materials and methods

### 2.1 Phylogenetic and protein-protein interaction (PPI) analysis

The protein sequences of human DBL family Rho GEFs (70 members) and human DOCK family Rho GEFs (11 members) were obtained from UniProt (https://www.uniprot.org/). The phylogenetic tree was constructed by MEGA7.0 software using neighbor-joining method with default parameters and 1000 bootstrap replicates [17]. The final tree containing the information of the domain organization was visualized by the online tool Evolview [18]. PPI networks were created by Search Tool for the Retrieval of Interacting Genes (STRING) (correlation score > 0.4) [19].

### 2.2 Oncomine database analysis

Oncomine (https://www.oncomine.org/) is an online mining platform by which the expression analyses on comparing the transcriptome data in most types of cancer with their corresponding normal tissues can be performed [20,21]. The mRNA expression levels of genes encoding Rho GEFS in NSCLC tissues, represented by LUAD and LUSC, as well as normal lung tissues were analyzed by Oncomine. In this study, *P*-value < 0.05, fold change > 2.0 and top 5% gene rank were selected as the thresholds. The resulting data were input into and visualized by Microsoft Office Excel 16.0 software (Microsoft Corporation, Redmond, CA).

### 2.3 Gene Expression Profiling Interactive Analysis (GEPIA) database analysis

GEPIA (http://gepia.cancer-pku.cn/) is an online tool that contains differential expression analysis between tumors and normal tissues based on The Cancer Genome Atlas (TCGA) and Genotype-Tissue Expression (GTEx) data [22]. To date, TCGA has produced RNA-Seq data including 9736 tumor samples across 33 cancer types, as well as data containing 726 adjacent normal tissues. The GTEx project contains RNA-Seq data for more than 8000 normal samples from unrelated donors. GEPIA integrates the information from cancer genomics big data for end users. The GEPIA database was used to validate the transcriptional profiles of Rho GEFs in patients with NSCLC.

### 2.4 UALCAN database analysis

UALCAN (http://ualcan.path.uab.edu/) is a web server that uses TCGA RNA-seq and clinical data from 31 cancer types [23]. This database provides a platform for identifying candidate biomarkers specific for tumor sub-groups. The UALCAN database was utilized to analyze the mRNA expression of normal tissues and NSCLC specimens from different sub-groups based on nodal metastasis status and tumor stages.

### 2.5 Kaplan–Meier Plotter database analysis

Kaplan–Meier Plotter (https://kmplot.com/) is an online database capable of assessing the effects of 54,000 genes on survival in 21 types of cancer. This system includes gene chip and RNA-seq data from the Gene Expression Omnibus (GEO), European Genome-Phenome Archive (EGA), and TCGA database. In this database, data of lung cancer are available [24]. The patient specimens were divided into high expression and low expression groups using the JetSet best probe set and auto-selected best cutoff [25]. Outlier arrays were excluded to control the array quality [26]. Overall survival (OS) and first progression (FP) were analyzed in patients with NSCLC. Log-rank *P*-value and hazard ratio (HR) with 95% confidence interval (CI) were calculated and displayed on the web server. The Kaplan–Meier Plotter database was used to evaluate the prognostic value of genes encoding Rho GEFs in NSCLC.

### 2.6 MEXPRESS database analysis

MEXPRESS (https://mexpress.be/) is an online data visualization tool for the visualization and integration of TCGA expression, DNA methylation and clinical data [27,28]. The MEXPRESS database was employed to investigate the promoter methylation status of selected genes in NSCLC specimens compared to normal tissues, and only CpG sites with statistically significant results were reported.

## 2.7 cBioPortal for Cancer Genomics (cBioPortal) database analysis

cBioPortal (http://cbioportal.org) is a web resource for the exploration, visualization and analysis of cancer genomics data [29]. The datasets selected in our study were LUAD (TCGA, Firehose Legacy, containing 580 samples) and LUSC (TCGA, Firehose Legacy, containing 503 samples). The platform for methylation sequencing information was the Illumina Human Methylation 450 (HM450). The correlation between the expression of the selected genes and DNA methylation in LUAD and LUSC was determined by cBioPortal. Moreover, the mutations of the selected genes and their relationship with gene expression were investigated using cBioPortal.

### 2.8 Gene Set Enrichment Analysis (GSEA)

GSEA was used to determine whether a defined gene set was expressed with significant differences under two different biological conditions [30]. The TCGA data regarding LUAD (n = 479) and LUSC (n = 501) patients were downloaded from the Genomic Data Commons (GDC) Data Portal website (https://portal.gdc.cancer.gov/). Subsequently, the patients were classified into two groups (high vs. low expression) for each selected gene, and the cutoffs were determined as medians. GSEA was conducted on the mRNA expression data of the selected genes using GSEA 4.0.3 software (http://software.broadinstitute.org/gsea/). A list of protein-coding genes of hallmark gene sets (H) from the Molecular Signatures Database (MSigDB) (https://www.gsea-msigdb.org/gsea/msigdb/) was obtained for GSEA analysis [31], with 1,000 permutations to calculate the normalized enrichment score (NES) and *P* values. Gene sets with a false discovery rate (FDR) of < 0.25 and *P* < 0.05 were regarded as significantly enriched gene sets.

### 2.9 Cell lines and cell culture

293 T cells, adenocarcinoma cell PC9 and squamous cell H1703 in human NSCLC were used in this study to verify the tumor-suppressive effect of ABR. The 293 T cells were cultured in Dulbecco’s modified Eagle’s medium (DMEM) with high glucose (Hyclone; GE Healthcare Life Sciences, Logan, UT, USA). PC9 and H1703 cells were cultured in Roswell Park Memorial Institute (RPMI) 1640 medium (Gibco; Thermo Fisher Scientific, Inc., Waltham, MA, USA). All media were supplemented with 10% fetal bovine serum (FBS Gibco; Thermo Fisher Scientific, Inc., Waltham, MA, USA), 100 U/mL penicillin and 100 mg/L streptomycin. All cell lines were incubated in a 5% CO_2_, 95% humidity incubator at 37°C.

### 2.10 Plasmid and stable-overexpression cell lines construction

The full-length sequences of ABR CF1: CCGCTCGAGGCCACCATGGAGCCGCTCAGCCACCGG and ABR CR1:GGAATTCCACGTCGGTGGAGAAGTACAG were amplified from 293 T cells with primers (NM_ 021962.5). Then ABR sequences were connected to pcDNA3.1 with a flag tag and subcloned into the pBOBI-IRES-EGFP-T2A-Puro vector. After the virus was packaged in 293 T cells using Lipofectamine 3000 (Life Invitrogen, USA), ABR and an empty vector were transfected into the target cell line lung cancer cells for overexpression. According to the fluorescence expression, the cells were screened with puromycin (1 μg/mL) for 4 to 6 days.

### 2.11 Protein extraction and Western blotting

Total protein was extracted from all cell lines with RIPA buffer including 100X Thermo Cocktail Protease inhibitor (Thermo Fisher Scientific, Inc., Waltham, MA, USA). The cell lysates were subjected to SDS-PAGE and transferred to a polyvinylidene fluoride (PVDF) membrane. The PVDF membrane was blocked with 5% fat-free milk and incubated with the primary antibody directed against ABR (Abcam, UK) at room temperature for 1 h. Subsequently, the PVDF membrane was washed with TBS-Tween 20 (TBST) for three times, incubated with a horseradish peroxidase (HRP) goat anti-Rabbit (IgG) secondary antibody (Abcam, UK) at room temperature for 1 h, and washed again with TBST for three times. The PVDF membrane was imaged using FluorChem M (ProteinSimple, CA, USA). After stripping, the PVDF membrane was re-probed with β-actin antibody (Proteintech Group, USA) and the HRP-conjugated Affinipure Goat Anti-Mouse IgG (H + L) (Proteintech Group, CA, USA) as the secondary antibody.

### 2.12 Cell proliferation assay

The cells were seeded into 96 well plates at a density of 10,000 cells per well. Cell viability was measured at 2h, 24 h, 48 h and 72 h after inoculation. The absorbance of 490 nm was measured at 37°C for 1 h after incubation with Cell Titer 96® AQueous One Solution Cell Proliferation Assay (MTS) (Promega, WI, USA). Results are representative of three independent experiments.

### 2.13 Colony formation assay

Cells were seeded at a density of 1,000 cells per well in 6-well plates and cultured at 37°C for colony formation. After 7 to 14 days, the cells were fixed in anhydrous formaldehyde for 15 min and stained with 0.1% (w/v) crystal violet at room temperature for 15 min. The colonies were photographed using a Fluor Chem R Imaging system (Protein Simple) and counted by Image J software. Results are representative of three independent experiments.

### 2.14 Cell migration assay

After starvation in serum-free medium for 12 h, the cells were seeded into a Transwell chamber at a density of 50,000 cells per well. The cells were cultured in serum-free or 10% FBS medium in a chamber. The cells were cultured in a 37°C and 5% CO_2_ incubator for 24 h. Finally, the cells were fixed in anhydrous formaldehyde for 15 min, stained with 0.1% (w/v) crystal violet for 15 min, and rinsed with PBS. After that, the cells were placed under a 200X microscope, photographed for 10 fields, and counted by Image J software. Results are representative of three independent experiments.

## 3. Results

Rho GEFs are conventionally known as activators of Rho GTPases, which promote carcinogenesis. To date, there is a lack of comprehensive understanding of the role of Rho GEFs in NSCLC. The aim of our study was to provide a more comprehensive understanding of Rho GEFs. We hypothesized that Rho GEFs can exert functions in addition to Rho GTPase activation in NSCLC. Our bioinformatic analysis and in vitro experiments confirmed our hypothesis for the Rho GEF ABR.

### 3.1 Structure and domains of Rho GEF family proteins

**Figure 1.**
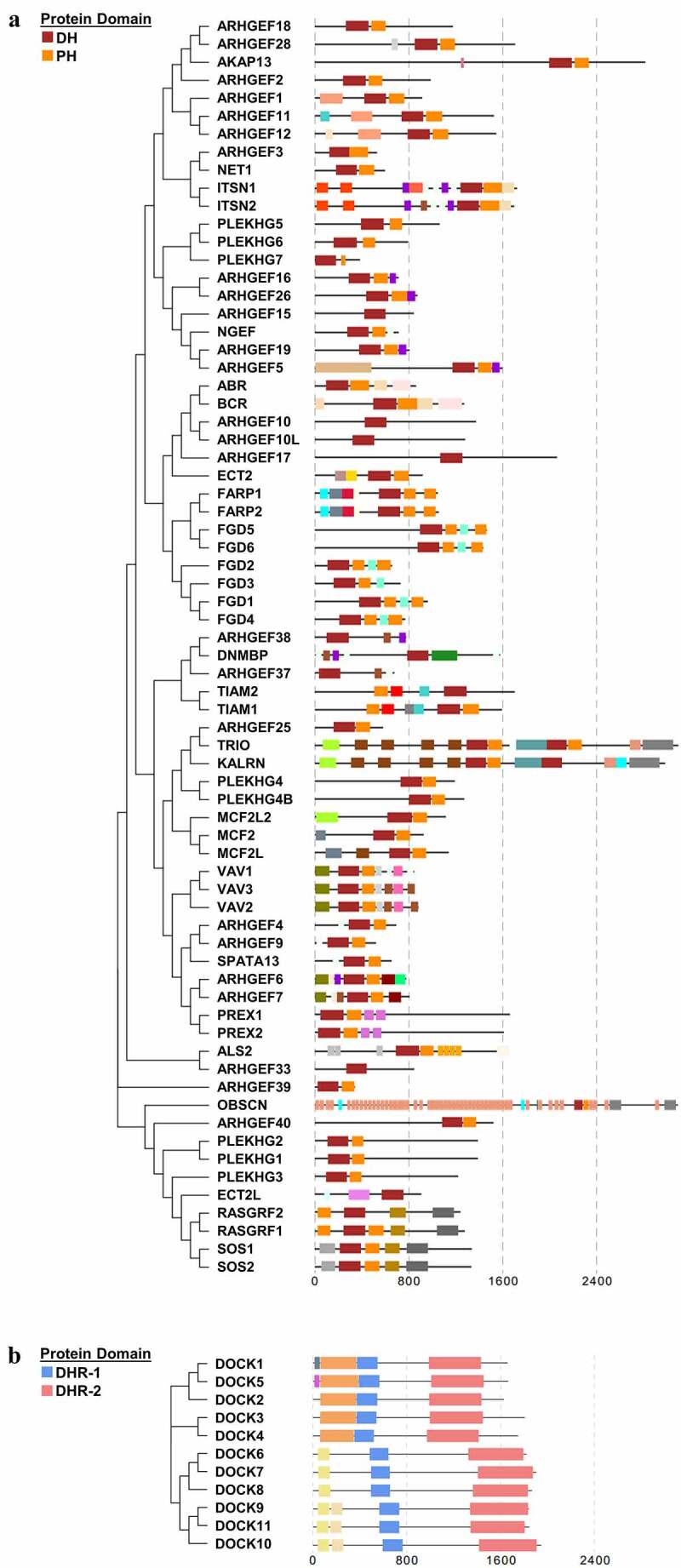
**The human DBL family and DOCK family of Rho GEFs**. (a) The 70 members of DBL family Rho GEFs and (b) 11 members of DOCK family Rho GEFs are arranged in phylogenetic tree by MEGA7.0 software, using the protein sequences obtained from UniProt. The protein domain architecture is visualized by Evolview. Each color represents one distinct structural domain. The DH, PH domains of DBL Rho GEF family and DHR-1, DHR2 domains of DOCK Rho GEF family with the corresponding colors are highlighted in the left panel. All proteins are visualized in the same scale except OBSCN. For other structural domains, please refer to FigureS1. DH: Dbl homology; PH: Pleckstrin homology; DHR-1: DOCK homology region 1; DHR-2: DOCK homology region

The human Rho GEFs are comprised of 81 members, which can be mainly divided into two families, namely DOCK family (Figure 1a) and DBL family (Figure 1b). The structures of Rho GEFs are various, but conserved domains have also been identified. Proteins of the DBL Rho GEFs were defined by the presence of conserved Dbl-homology (DH) domains. The DBL family of Rho GEFs contains 70 members with 72 DH domains, among which TRIO and KALIRIN possess two DH domains, and the other members all contain one DH domain, which is responsible for stimulating guanine nucleotide exchange. In addition to the DH domain, most DBL Rho GEFs contain a pleckstrin-homology (PH) domain following the DH domain. The PH domains are able to bind to phospholipids and are essential to properly mediate the localization of DBL Rho GEFs to their action sites via lipid binding. Proteins of the DOCK Rho GEFs were classified by the presence of DOCK-homology region 1 (DHR-1) and DOCK-homology region 1 (DHR-2) domains. Similar to the DH and PH domains, DHR-2 domains are responsible for guanine nucleotide exchange, and DHR-1 domains interact with phospholipids, which aid in targeting DOCK Rho GEFs to the plasma membrane. Apart from the DH, PH, DHR-1, and DHR-2 domains, other domains of Rho GEFs, such as Src homology 2 (SH2) and Src homology 3 (SH3) domains, are important in mediating protein-protein interactions, protein-lipid interactions, and messenger binding. In order to highlight the highly conserved domains of Rho GEFs, the details of the other domains displayed in Figure 1 are illustrated in Supplementary FigureS1. The PPIs of the Rho GEFs are demonstrated in **Supplementary Fig. S2**,

### 3.2 The expression levels of Rho GEFs are altered in lung cancer.

**Figure 2.**
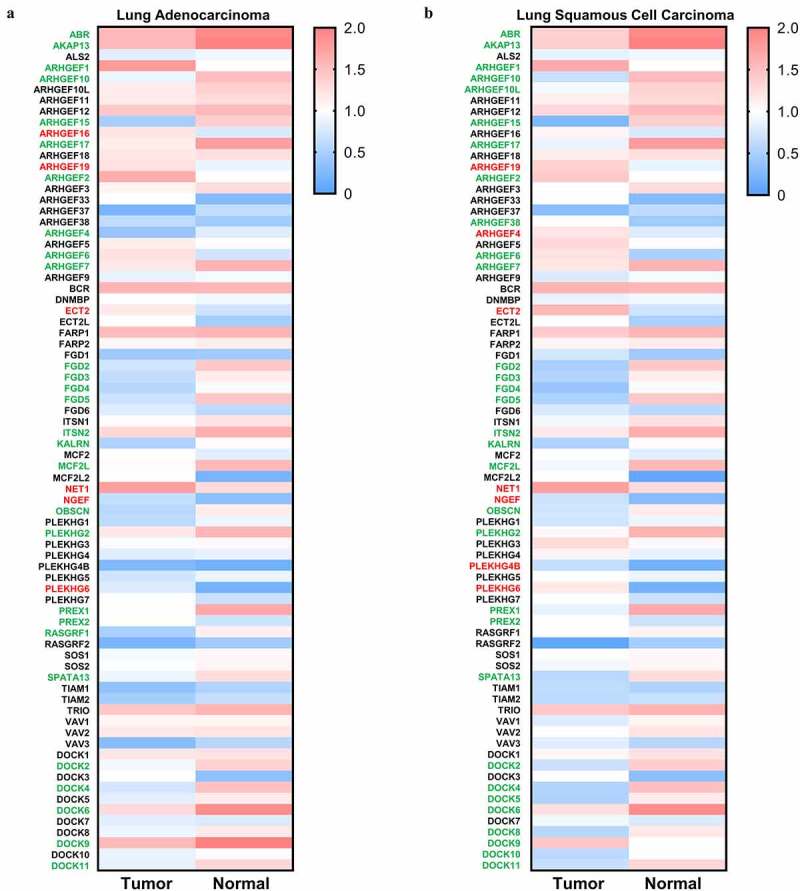
**Expression of Rho GEFs in NSCLC**. The expression levels of Rho GEFs in NSCLC, represented by LUSC and LUAD, are analyzed by GEPIA database. (a) The median expression of Rho GEFs in LUAD compared to normal tissues. (b) The median expression of Rho GEFs in LUAD compared to normal tissues. The transcripts per million (TPM) of genes are normalized by log_10_(TPM). Gene name with red (higher expression compared to normal tissue) and green (lower expression compared to normal tissue) color indicates *P* < 0.05 for their expressions in tumors compared to normal tissues

Oncomine database analysis was performed to investigate the altered mRNA expression in lung cancer. There are 43 genes encoding DBL GEFs (Supplementary FigureS3A) and 7 genes (Supplementary FigureS3B) encoding DOCK GEFs showing remarkable alterations of mRNA expression levels in lung cancer compared to normal tissues. For the DBL family of Rho GEFs, 24 genes were downregulated in lung cancer, whereas 13 genes were upregulated, and the other 6 genes showed contradictory results of aberrant expression in different datasets. For the DOCK family of Rho GEFs, 6 genes were underexpressed in lung cancer, while only 1 gene was identified to be overexpressed. Moreover, GEPIA database analysis was used to verify the altered mRNA expression of genes encoding Rho GEFs in NSCLC. Due to the fact that gene expression patterns can vary in LUAD and LUSC, the following analyses were performed independently in LUAD and LUSC [32]. Most of the genes encoding DBL Rho GEFs were downregulated in LUAD (Figure 2a) and LUSC (Figure 2b). For all of the DOCK Rho GEFs except DOCK7, the median expression of genes was also decreased in LUAD (Figure 2a) and LUSC (Figure 2b) compared to normal samples. Furthermore, the UALCAN and Kaplan Meier Plotter database analyses for all genes encoding Rho GEFs were performed (data not shown). The genes showing contradictory effects within or across different databases were excluded. Eventually, ABR and PREX1 in the DBL family of Rho GEFs, as well as DOCK2 and DOCK4 in the DOCK family of Rho GEFs, were selected for this study.

**Figure 3.**
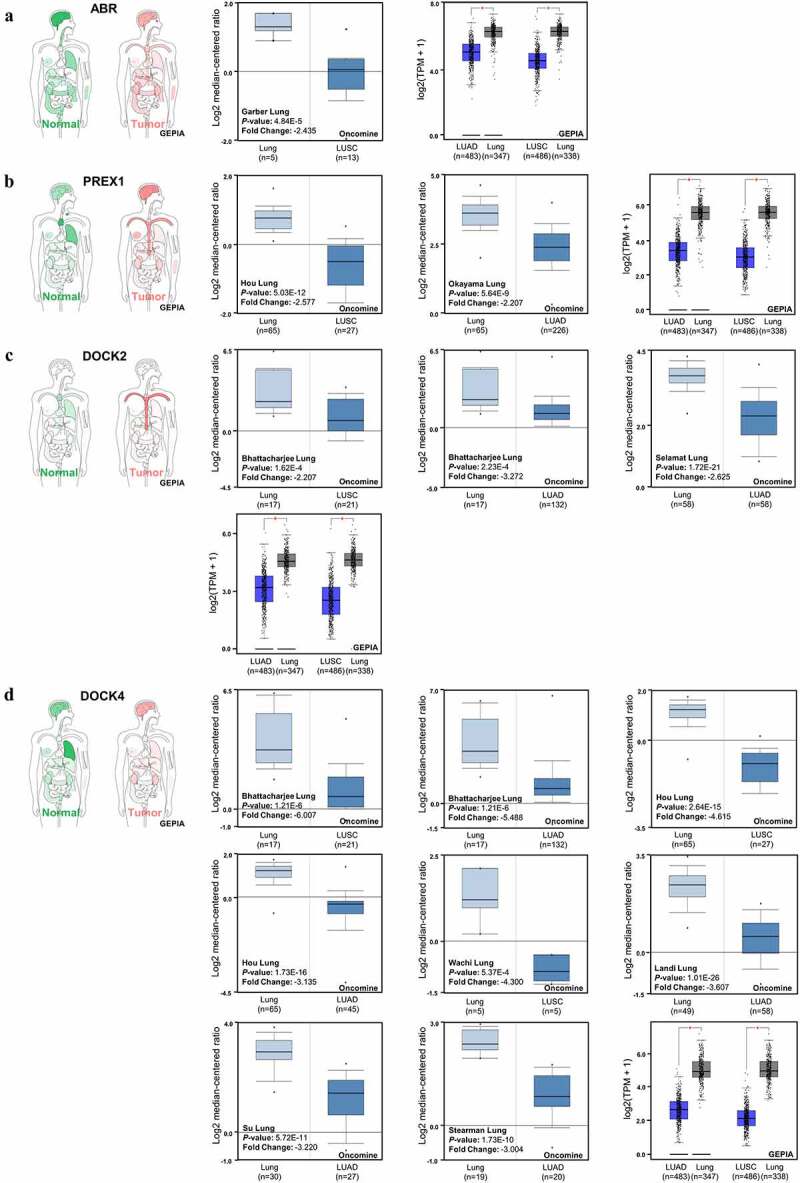
**Expression of ABR, PREX1, DOCK2 and DOCK4 in NSCLC**. (a) The body map of various organs (GEPIA), the cohort of Garber Lung (Oncomine), and the LUSC and LUAD patient specimens with normal tissues for the expression of ABR (GEPIA) are illustrated. (b) The body map (GEPIA), the cohorts of Hou Lung and Okayama Lung (Oncomine), and the LUSC and LUAD patient samples with normal tissues for PREX1 expression (GEPIA) are demonstrated. (c) The body map including different organs (GEPIA), the cohorts of Bhattacharjee Lung and Selamat Lung (Oncomine), and the LUSC and LUAD patient samples with normal tissues for DOCK2 expression (GEPIA) are shown. (d) The body map (GEPIA), the cohorts of Bhattacharjee Lung, Hou Lung, Wachi Lung, Landi Lung, Su Lung and Stearman Lung (Oncomine), and the LUSC and LUAD patient samples with normal tissues for DOCK4 expression (GEPIA) are illustrated. Red: tumor tissue. Green: normal tissue. The deeper color in the body map represents higher expression.*P < 0.05

### 3.3 ABR, PREX1, DOCK2 and DOCK4 are downregulated in NSCLC.

In order to obtain an overview of the expression patterns of ABR in various tumor types and in the corresponding normal tissues, a body map was generated by GEPIA (Figure 3a). Compared to normal lung tissue, the expression of ABR is lower in lung cancer. To further validate the downregulation of ABR in lung cancer, specific datasets from Oncomine analysis are illustrated (Figure 3a). From the Garber Lung dataset, the expression of ABR in LUSC was markedly lower than that in normal lung tissue (*P* < 0.001, fold change −2.435), while the datasets of LUAD with statistical significance are lacking in the Oncomine database. Therefore, the GEPIA database was used to confirm our results. From the GEPIA database analysis of both LUAD and LUSC, ABR was significantly downregulated in tumors (*P* < 0.05). The downregulation of PREX1 (Figure 3b), DOCK2 (Figure 3c), and DOCK4 (Figure 3d) was also validated by Oncomine and GEPIA database analysis. These results imply that ABR, PREX1, DOCK2, and DOCK4 can function as tumor suppressors in NSCLC, while more specific data on tumor subgroups and the corresponding survival data are needed for confirmation. Consequently, the expression levels of the selected genes in the tumor subgroups divided by nodal metastasis status or individual stages were investigated.

From the UALCAN database analysis, ABR (Supplementary Figure 4a), PREX1 (Supplementary Figure 4c), DOCK2 (Supplementary Figure 4e), and DOCK4 (Supplementary Figure 4g) were significantly downregulated in each nodal metastasis status (N0, N1, N2, and N3) of LUAD and LUSC when compared to normal tissues. In addition, the expression of ABR (Supplementary Figure 4b), PREX1 (Supplementary Figure 4d), DOCK2 (Supplementary figure 4f), and DOCK4 (Supplementary Figure 4h) were observably lower in each stage (stage 1, stage 2, stage 3 and stage 4) of LUAD and LUSC compared to normal samples. Although the median expression of the selected genes generally decreased as nodal metastasis status or tumor stage advanced, the majority of these data showed no statistical significance. Taken together, these consistent results confirm that ABR, PREX1, DOCK2, and DOCK4 are notably downregulated in NSCLC, and their prognostic values were subsequently investigated.

**Figure 4.**
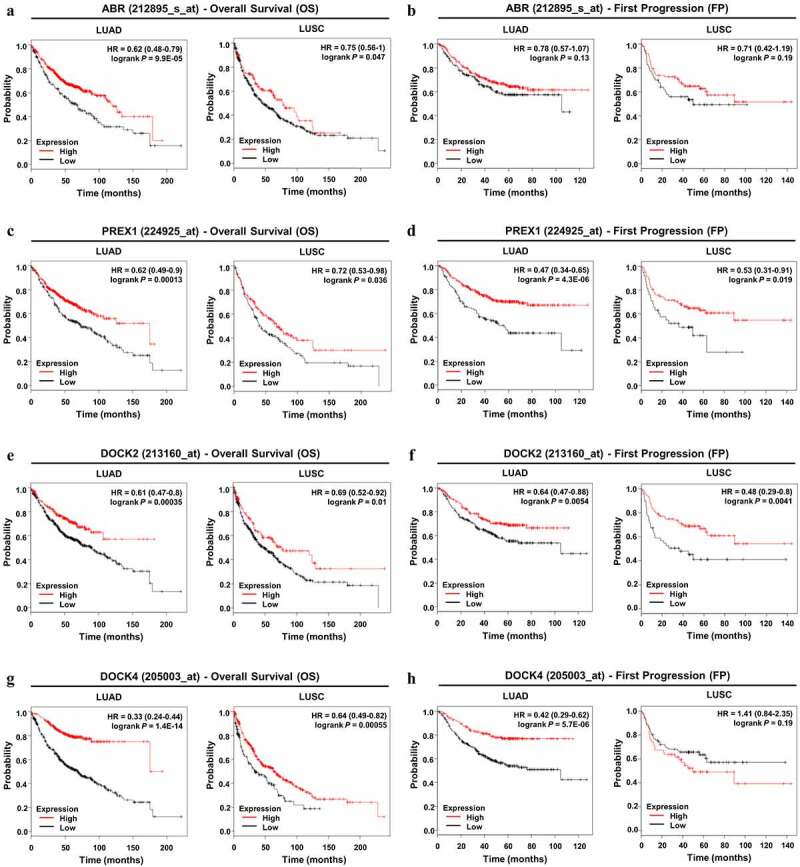
**Prognostic value of ABR, PREX1, DOCK2 and DOCK4 expression in NSCLC. (A, C, E, G)** Survival curves referring to OS are plotted for LUAD and LUSC patients. **(B, D, F, H)** Survival curves with regard to FP are generated for patients with LUAD and LUSC. Log-rank *P* values and HRs with 95% CIs are displayed. FP: first progression; HR: hazard ratio; OS: overall survival

### 3.4 Downregulations of ABR, PREX1, DOCK2 and DOCK4 are prognostic factors for NSCLC.

Using the Kaplan–Meier Plotter database, survival analysis was performed to determine the prognostic value of the selected genes in NSCLC patients. With regard to overall survival, ABR (Figure 4a), PREX1 (Figure 4c), DOCK2 (Figure 4e), and DOCK4 (Figure 4g) all demonstrated significant values in the prognosis of LUAD and LUSC patients, and a lower expression of these genes predicted poor OS in patients. For LUAD patients, the expression of DOCK4 was more significantly correlated with OS (HR = 0.33; 95% CI: 0.24–0.44; *P* = 1.4E-14) compared to that of ABR, PREX1, and DOCK2. In LUSC patients, DOCK4 expression was also more markedly correlated with OS (HR = 0.64; 95% CI: 0.49–0.82; *P* = 0.00055) than ABR, PREX1, and DOCK2 expression. However, for the prognosis of tumor progression, ABR showed no significance in both LUAD and LUSC patients (Figure 4b). In addition, lower DOCK4 expression was associated with earlier FP in LUAD patients, but not in LUSC patients (Figure 4h). The downregulation of PREX1 (Figure 4d) and DOCK2 (figure 4f) were significantly correlated with earlier tumor progression in LUSC and LUAD patients. Among the four Rho GEFs, DOCK4 predicted the progression in LUAD patients with a higher significance (HR = 0.42; 95% CI: 0.29–0.62; *P* = 5.7E-06), while DOCK2 more observably predicted LUSC progression (HR = 0.48; 95% CI: 0.29–0.8; *P* = 0.0041). Collectively, through the analysis using Oncomine, GEPIA, UALCAN, and Kaplan–Meier Plotter databases, consistent results highlight the downregulation of ABR, PREX1, DOCK2, and DOCK4 in NSCLC, and they notably predict the poor prognosis of NSCLC patients.

#### 3.5 Promoter methylation levels contribute to the aberrant expression of ABR, PREX1, DOCK2, and DOCK4 in NSCLC

**Figure 5.**
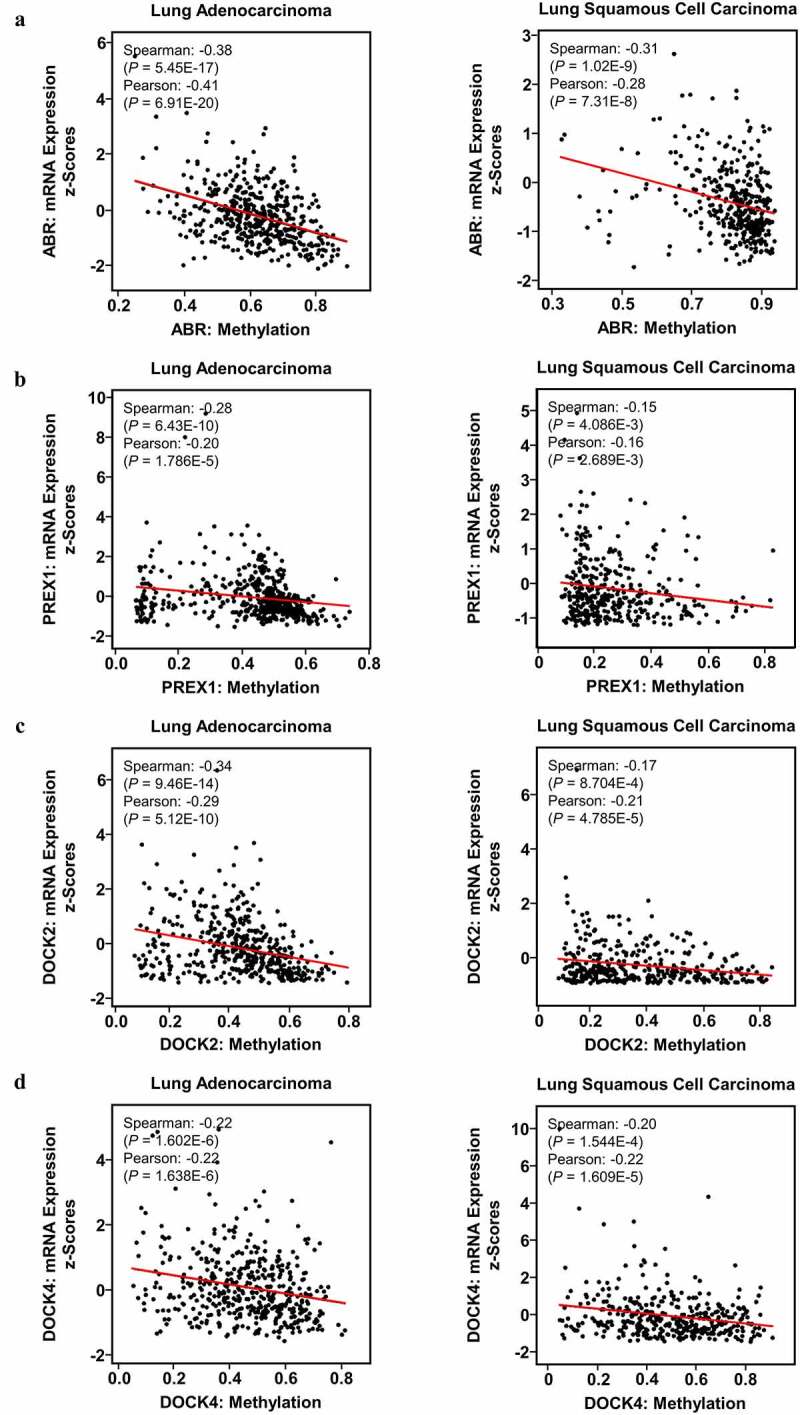
**Impact of DNA methylation on ABR, PREX1, DOCK2 and DOCK4 expression in NSCLC**. Visualization of TCGA data for the promoter region methylation and mRNA expression of (a) ABR, (b) PREX1, (c) DOCK2 and (d) DOCK4 in LUAD and LUSC, compared to those in normal tissues using MEXPRESS. The correlation of (e) ABR, (f) PREX1, (g) DOCK2 and (h) DOCK4 expression with promoter methylation is analyzed by cBioPortal database. Spearman’s correlation analysis and Pearson’s correlation analysis are performed in the database. The regression line is used to illustrate the correlation trend

DNA methylation in promoter regions is important for modulating gene expression by inducing stable epigenetic inhibition of gene expression. Therefore, whether methylation is correlated with the expression of ABR, PREX1, DOCK2, and DOCK4 was examined. cBioPortal database analysis revealed that the promoter methylation levels of ABR (*P* = 6.91E-20, r = −0.41 for LUAD; *P* = 7.31E-8, r = −0.28 for LUSC) (Figure 5a), PREX1 (*P* = 1.786E-5, r = −0.20 for LUAD; *P* = 2.689E-3, r = −0.16 for LUSC) (Figure 5b), DOCK2 (*P* = 5.12E-10, r = −0.29 for LUAD; *P* = 4.785E-5, r = −0.21 for LUSC) (Figure 5c) and DOCK4 (*P* = 1.638E-6, r = −0.22 for LUAD; *P* = 1.609E-5, r = −0.22 for LUSC) (Figure 5d) were negatively correlated with mRNA expression in NSCLC. MEXPRESS database revealed a large number of probes that were significantly associated with the expressions of ABR, PREX1, DOCK2 and DOCK4 (Supplementary FigureS5). The above results suggest that the hypermethylation of ABR, PREX1, DOCK2, and DOCK4 is not only identified in NSCLC in comparison to normal tissues, but is also negatively correlated with their mRNA expression in NSCLC.

### 3.6 Analysis of ABR, PREX1, DOCK2, and DOCK4 mutations in NSCLC.

Genetic alterations of ABR, PREX1, DOCK2, and DOCK4 were studied by the cBioPortal database. The proportion and distribution of TCGA samples with genetic alterations in LUAD (Supplementary FigureS6A) and LUSC (Supplementary FigureS6B) are shown, and DOCK2 possesses the highest proportion of genetic alterations in both LUAD (13%) and LUSC (10%) among the four selected Rho GEFs. The locations of the mutations are further illustrated in Supplementary FigureS6C and Supplementary FigureS6D. The majority of the mutations in the four selected Rho GEFs were missense mutations, and several mutations were located in the regions encoding the conserved domains of Rho GEFs. However, the mutations of the selected genes have no statistically significant impact on the mRNA expression in LUAD (Supplementary FigureS6E) and LUSC (Supplementary FigureS6F).

**Figure 6.**
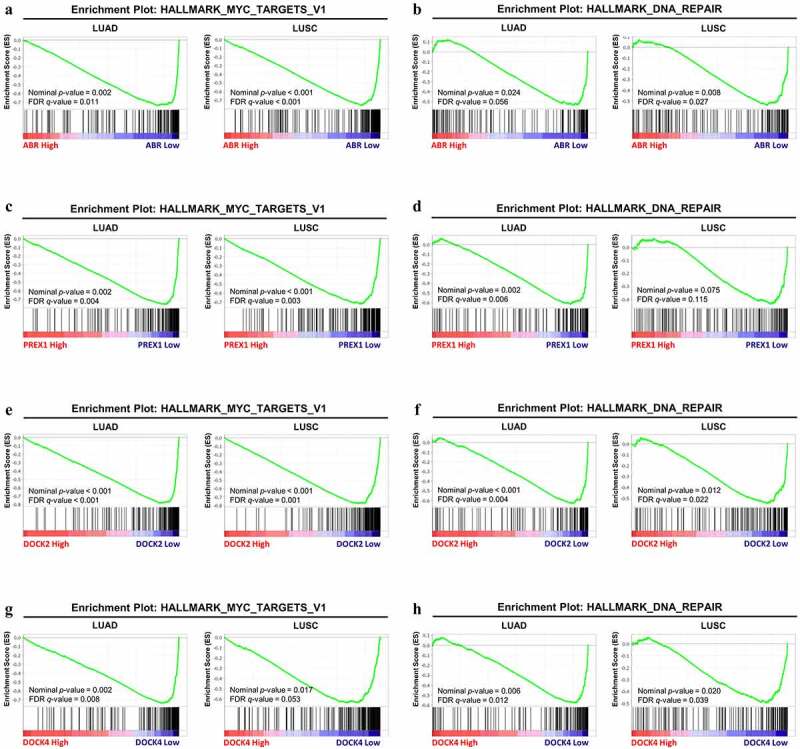
**GSEA analysis between the high-expression group and low-expression group in ABR, PREX1, DOCK2 and DOCK4 for NSCLC**. The correlation between the enrichment of MYC signaling gene set and (a) ABR, (c) PREX1, (e) DOCK2 and (g) DOCK4 expression in LUAD and LUSC. The association between the expression of (b) ABR, (d) PREX1, (f) DOCK2 and (h) DOCK4 expression with DNA repair gene set enrichment. The barcode plot demonstrates gene positions in each gene set. The horizontal bar indicates positive (red) and negative correlation (blue) with gene expression. FDR: false discovery rate

#### 3.7 GSEA identifies potential mechanisms by which ABR, PREX1, DOCK2, and DOCK4 regulate NSCLC development and progression

Based on the data from multiple analyses, the lower expression of ABR, PREX1, DOCK2, and DOCK4 can lead to NSCLC development and progression, and therefore, GSEA by TCGA data was employed to explain the underlying mechanisms. The expression matrix of NSCLC patients is divided into high-expression and low-expression groups based on the median expression levels of ABR, PREX1, DOCK2, and DOCK4, respectively. GSEA revealed that, in LUAD and LUSC, patients with lower expression of ABR (nominal *P* = 0.002, FDR = 0.011 for LUAD; nominal *P* < 0.001, FDR < 0.001 for LUSC) (Figure 6a), PREX1 (nominal *P* = 0.002, FDR = 0.004 for LUAD; nominal *P* < 0.001, FDR = 0.003 for LUSC) (Figure 6c), DOCK2 (nominal *P* < 0.001, FDR < 0.001 for LUAD; nominal *P* < 0.001, FDR = 0.001 for LUSC) (Figure 6e) and DOCK4 (nominal *P* = 0.002, FDR = 0.008 for LUAD; nominal *P* = 0.017, FDR = 0.053 for LUSC) (Figure 6g) have a higher expression of genes associated with MYC signaling, which are essential for the development of lung cancer [33–35]. Moreover, our results also revealed that the DNA repair pathway was significantly enriched under the downregulation of ABR (nominal *P* = 0.024, FDR = 0.056) (Figure 6b), PREX1 (nominal *P* = 0.002, FDR = 0.006) (Figure 6d), DOCK2 (nominal *P* < 0.001, FDR = 0.004) (figure 6f), and DOCK4 (nominal *P* = 0.006, FDR = 0.012) in LUAD (Figure 6h). Similarly, the DNA repair pathway enrichment was identified under ABR (nominal *P* = 0.008, FDR = 0.027) (Figure 6b), DOCK2 (nominal *P* = 0.012, FDR = 0.022) (figure 6f), and DOCK4 (nominal *P* = 0.020, FDR = 0.039) (Figure 6h) downregulation in LUSC. However, the DNA repair pathway was not enriched in the PREX1-low-expression group in LUSC (nominal *P* = 0.075, FDR = 0.115) (Figure 6d). The details of the data are summarized in Table 1.

**Table 1.** Gene Set Enrichment Analysis (GSEA) of gene sets significantly enriched in LUAD and LUSC

Tumor Types	Genes Encoding GEFs	Enriched Gene Sets	Size	ES	NES	Nominal *p*-value	FDR *q*-value	FWER *p*-value
LUAD	ABR	HALLMARK_MYC_TARGETS_V1	199	−0.73912	−2.03656	0.002016	0.011191	0.027
		HALLMARK_DNA_REPAIR	150	−0.53791	−1.77365	0.023762	0.056209	0.191
	PREX1	HALLMARK_MYC_TARGETS_V1	199	−0.75718	−2.15914	0.001957	0.00364	0.007
		HALLMARK_DNA_REPAIR	150	−0.61628	−2.06687	0.00198	0.00618	0.022
	DOCK2	HALLMARK_MYC_TARGETS_V1	199	−0.7828	−2.27298	0	7.28E-04	0.002
		HALLMARK_DNA_REPAIR	150	−0.63067	−2.13108	0	0.004392	0.016
	DOCK4	HALLMARK_MYC_TARGETS_V1	199	−0.73671	−2.02493	0.001938	0.008172	0.032
		HALLMARK_DNA_REPAIR	150	−0.57858	−1.94703	0.005725	0.012478	0.056
LUSC	ABR	HALLMARK_MYC_TARGETS_V1	199	−0.7608	−2.25999	0	7.39E-04	0.003
		HALLMARK_DNA_REPAIR	150	−0.54108	−1.88272	0.008403	0.02707	0.116
	PREX1	HALLMARK_MYC_TARGETS_V1	199	−0.72201	−2.17652	0	0.003185	0.009
		HALLMARK_DNA_REPAIR	150	−0.43784	−1.51756	0.075397	0.115496	0.491
	DOCK2	HALLMARK_MYC_TARGETS_V1	199	−0.77524	−2.279	0	0.001378	0.002
		HALLMARK_DNA_REPAIR	150	−0.54779	−1.92649	0.012097	0.022362	0.091
	DOCK4	HALLMARK_MYC_TARGETS_V1	199	−0.63924	−1.9451	0.016736	0.05325	0.077
		HALLMARK_DNA_REPAIR	150	−0.49595	−1.81377	0.02004	0.039442	0.183

ES: enrichment score; NES: normalized enrichment score; FDR: false discovery rate; FWER: family-wise error rate.

#### 3.8 ABR shows tumor suppression effect in proliferation, migration, and cloning ability in PC9 and H1703 cells

**Figure 7.**
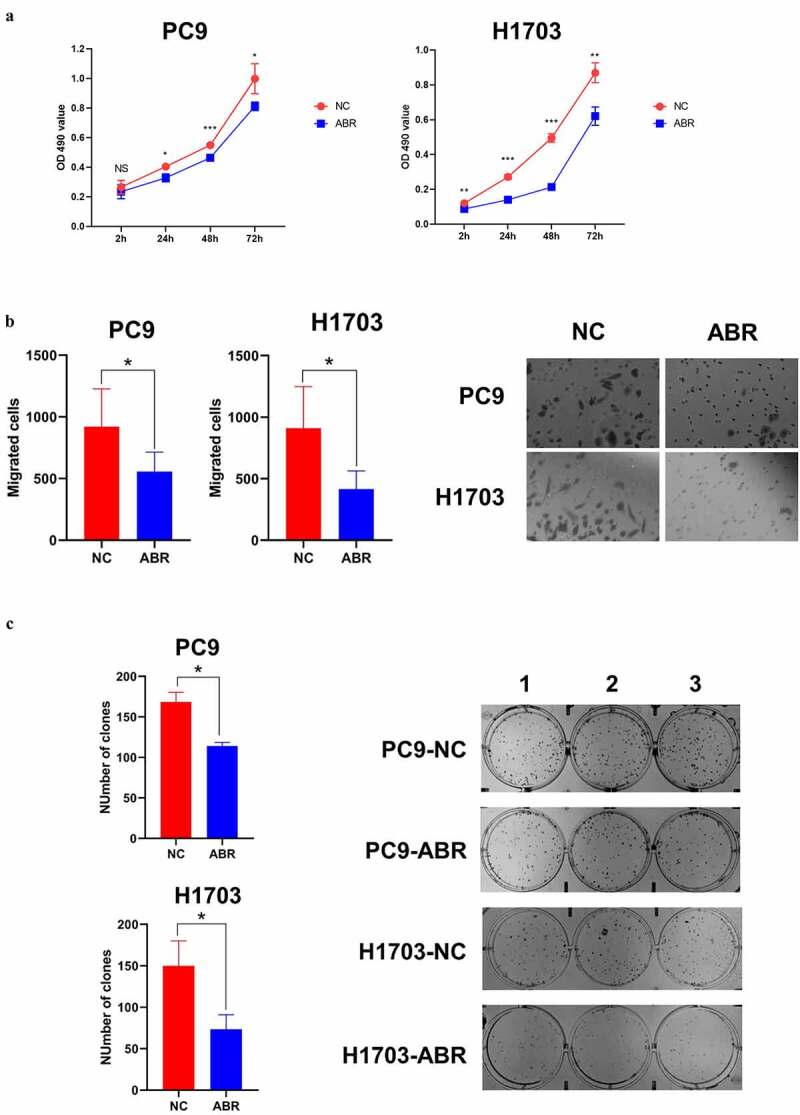
**ABR shows tumor suppression effect in proliferation, migration and cloning ability in PC9 and H1703 cells**. The cell proliferation experiment of PC9 and H1703 cell lines with overexpression of ABR (a). The migration capacity of PC9 and H1703 cells under ABR overexpression is shown, and the right panel illustrates tumor cells that invaded the chamber (b). The cloning formation ability of PC9 and H1703 cells under ABR overexpression, and the right panel demonstrates the colonies formed in each well (c). NC: control group with transfection by empty vector. ABR: ABR overexpressed group. NS: non-significant, * *P* < 0.05, ** *P* < 0.01, *** *P* < 0.001

In addition to analyzing the tumor-suppressive effects of ABR, PREX1, DOCK2, and DOCK4 through bioinformatics, we further verified the role of ABR in proliferation, migration, and cloning ability in PC9 and H1703 cells experimentally. Cell proliferation experiments of PC9 and H1703 cell lines with overexpression of NC and ABR1 showed that overexpression of ABR inhibited cell proliferation in both cell types (Figure 7a). The migration capacity of ABR overexpression was measured in PC9 and H1703 cells, which showed that ABR presented a tumor-suppressive effect when compared to the NC group (Figure 7b). Cloning ability was also tested and analyzed, and ABR overexpression showed a suppressive effect in the number of clones in both PC9 and H1703 cells (Figure 7c). The above results suggest that the overexpression of ABR, as a representative of Rho GEFs, is prone to exert a tumor-suppressive effect in NSCLC.

## 4. Discussions

Rho GEFs are known for their critical roles as molecular switches in activating Rho GTPases, and therefore function as regulators of various diseases not limited to cancer [5,6]. ABR, as indicated by its name, was first identified as a breakpoint cluster region (BCR)-related protein that shares high homology with BCR in humans (68% amino acid identity) [36]. ABR maintains the normal reactivity of the innate immune system, and the altered function of ABR can lead to the development of leukemia [37,38]. PREX1 was first discovered in the cytosol of neutrophils [39]. PREX1 is important in regulating reactive oxygen species (ROS) production, migration, and chemotaxis of neutrophils [40,41]. Elevated expression of PREX1 has been associated with the development of melanoma, prostate cancer, and breast cancer [42–44]. DOCK2 expression was initially deemed to be restricted to hematopoietic cells [45]. Although it is predominantly expressed in lymphocytes and hematopoietic tissues, recent research has revealed the tumor-promoting role of DOCK2 in lymphoma and colorectal cancer [46–48]. Distinct from the identification of ABR, PREX1 and DOCK2 in non-cancer cells, DOCK4 identification was initially reported in osteosarcoma cells, in which DOCK4 was deleted during tumor progression [49]. However, DOCK4 has also been reported to promote breast cancer development and is associated with bone metastasis [50,51]. To date, the roles of ABR, PREX1, DOCK2, and DOCK4 in lung cancer are largely unknown, and the underlying mechanisms remain to be explored.

Through the integration of data from multiple databases, our study demonstrated that ABR, PREX1, DOCK2, and DOCK4 are downregulated in NSCLC. In addition, NSCLC subgroups with higher nodal metastasis levels or cancer stages generally have lower median expression of ABR, PREX1, DOCK2, and DOCK4, whereas statistical significance for the comparison among these subgroups remains to be verified with a larger number of samples. Notably, the downregulation of ABR, PREX1, DOCK2, and DOCK4 are associated with poor overall survival of NSCLC patients, suggesting that these four novel Rho GEFs can serve as promising biomarkers for predicting the OS of patients. For the prognostic value for disease progression, only PREX1 and DOCK2 were statistically significant in both LUAD and LUSC patients. Although DOCK4 is not a candidate marker for predicting the disease progression in LUSC patients, its higher expression is markedly correlated with delayed tumor progression in LUAD patients. Hence, ABR, PREX1, DOCK2, and DOCK4 downregulation are characteristic of NSCLC, and they are promising for predicting the prognosis of NSCLC patients.

Gene expression and repression within cancer cells can be controlled by the epigenetic mechanisms of DNA methylation, which is the interaction between genes and phenotypes without causing mutations in the DNA sequence [52,53]. DNA methylation involves the covalent addition of methyl groups to the C-5 position of cytosine rings, especially in a CpG dinucleotide [54]. In mammals, approximately 70% of the promoters are rich in unmethylated CpG [55]. Hypermethylation of CpG sites in promoters is typically associated with gene silencing at the transcriptional levels [56]. In lung cancer, DNA hypermethylation of tumor suppressors represents a hallmark and an early event in tumorigenesis [57]. Our study revealed that NSCLC samples from TCGA database contain higher methylation levels in the promoters of ABR, PREX1, DOCK2 and DOCK4 genes compared to the normal samples. Moreover, in NSCLC specimens, elevated levels of promoter methylation were markedly associated with lower expression of ABR, PREX1, DOCK2, and DOCK4. Thus, DNA hypermethylation might contribute to the downregulation of ABR, PREX1, DOCK2, and DOCK4 in NSCLC, and the methylation profiles of the four key Rho GEFs may be novel biomarkers for lung cancer screening.

In addition to epigenetic alterations, gene mutations can also affect gene expression levels [58]. The majority of mutations for ABR, PREX1, DOCK2, and DOCK4 in NSCLC are missense and truncating mutations, which means a change of a single amino acid into another and a change in the DNA that can shorten the protein, respectively. Our results illustrate that the expression of ABR, PREX1, DOCK2, and DOCK4 in NSCLC are not correlated with mutations. It should be noted that loss-of-function or gain-of-function mutations can lead to potential inhibitory or tumorigenic effects [59]. From our analysis, a few mutations occurred in the RHOGEF (DH), PH, DOCK-C2 (DHR-1) and DHR-2 domains, indicating that the critical functions of Rho GEF can be altered by the mutations, and this is yet to be confirmed.

Mechanistically, the downregulation of ABR, PREX1, DOCK2 and DOCK4 might upregulate MYC signaling and DNA repair pathways, as identified by GSEA using TCGA data. The MYC oncogene encodes a transcription factor that triggers gene expression in cancer cells [60]. MYC signaling is implicated in the pathogenesis of most human cancers, and its deregulation is correlated with poor survival of patients [61]. MYC activation is associated with many features of cancer, including protein synthesis, proliferation and altered cellular pathways [61,62]. MYC upregulation is detected in >40% of NSCLC cases and is related to the loss of cell differentiation and tumor progression [63,64]. Therefore, ABR, PREX1, DOCK2, and DOCK4 downregulation could lead to the development of NSCLC via upregulation of the MYC protein expression and its downstream targets.

In clinical practice, chemotherapy and radiotherapy are the gold standards for the treatment of patients with lung cancer, and they have largely prolonged the survival of patients [65]. The therapeutic effects of platinum-based chemotherapeutic drugs and ionizing radiation are mediated by DNA damage [66–68]. Nevertheless, enhanced DNA repair mechanisms counteract the therapeutic benefits to patients, thereby leading to poor patient survival. Hence, the downregulation of ABR, PREX1, DOCK2, and DOCK4 promotes cancer development and leads to a poor prognosis by activating MYC and DNA repair signaling pathways in NSCLC, and further *in vitro* and *in vivo* studies are necessary to further confirm their association. Moreover, agents targeting DNA damage repair mechanisms have shown promise in NSCLC clinical models [67]. These four key Rho GEFs are also promising as biomarkers for predicting the response to DNA-damage regimens.

In spite of the conventional perspective that Rho GEFs are activators of Rho GTPases, based on comprehensive bioinformatic analysis and *in vitro* validation, our study unexpectedly but reasonably revealed that the Rho GEF ABR is a tumor suppressor in NSCLC. In addition to the above findings, it should be noted that the following evidence supports our results. ABR contains a Rho GAP domain in addition to its Rho GEF domain, functioning as a dual Rho GEF/GAP [69]. In addition, the prominent expression of ABR suggests its correlation with lymphocyte infiltration via cytokine secretion in NSCLC tissues. Tumor-infiltrating lymphocytes can attack and eliminate tumor cells, and therefore contribute to a better prognosis in patients with NSCLC [70,71]. For future studies, researchers should not simply regard all Rho GEFs as tumor promoters because of their involvement in activating Rho GTPases. Instead, the functions of Rho GEFs, other than the activation of Rho GEFs in cancers, should be noted.

## 5. Conclusion

In conclusion, our study identified that ABR, PREX1, DOCK2 and DOCK4 can serve as promising biomarkers for predicting the prognosis of NSCLC patients. The downregulation of ABR, PREX1, DOCK2 and DOCK4 in NSCLC can be induced by promoter methylation, and their methylation profiles might be potential indicators for lung cancer screening. The downregulation of the four key genes might enhance the tumorigenic MYC signaling and the DNA repair pathway. Our *in vitro* studies confirmed the role of ABR as a tumor suppressor in NSCLC cells. Future studies on methylation, regulation patterns and specific mechanisms are required.

## Supplementary Material

Supplemental Material

## Abbreviations

ABR,Active breakpoint cluster region-related; BCR, Breakpoint cluster region; CDC42, Cell division control protein 42 homolog; CI, Confidence interval; DH, Dbl-homology; DOCK, Dedicator of cytokinesis; DBL, Diffuse B-cell lymphoma; DHR, DOCK-homology region; EGA, European Genome-Phenome Archive; FDR, False discovery rate; FP, First progression; GDI, GDP-dissociation inhibitor; GEO, Gene Expression Omnibus; GEPIA, Gene Expression Profiling Interactive Analysis; GSEA, Gene Set Enrichment Analysis; GDC, Genomic Data Commons; GTEx, Genotype-Tissue Expression; GAP, GTPase-activating protein; GEF, Guanine nucleotide exchange factor; GDP, Guanosine diphosphate; GTPases, Guanosine triphosphatases; GTP, Guanosine triphosphate; HR, Hazard ratio; LUAD, Lung adenocarcinoma; LUSC: Lung squamous cell carcinoma; MSigDB, Molecular Signatures Database; NES, Normalized enrichment score; OS, Overall survival; PREX1, Phosphatidylinositol 3,4,5-trisphosphate-dependent Rac exchanger 1; PH, Pleckstrin-homology; RHOA, Ras homolog family member A; RAC1, Ras-related C3 botulinum toxin substrate 1; SH, Src homology; TCGA, The Cancer Genome Atlas.
