# Supplementary figures and images for: Role of Rho guanine nucleotide exchange factors in non-small cell lung cancer
Source: Bioengineered. 2021 Dec 2;12(2):11169–87. doi: 10.1080/21655979.2021.2006519 (PMC8810164; doi:10.1080/21655979.2021.2006519)

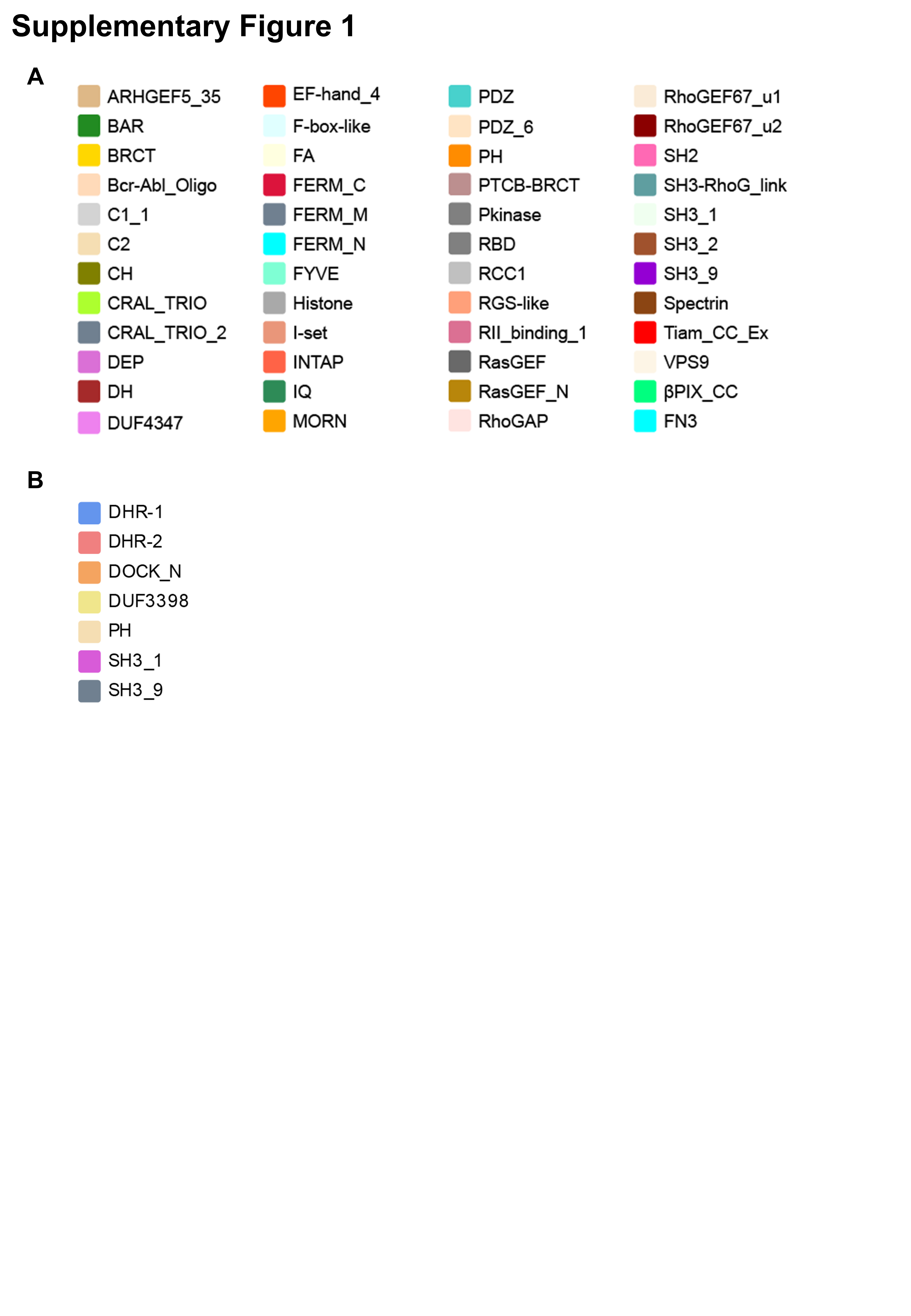

Supplement: Supplemental Material [file KBIE_A_2006519_SM0761.zip › supplementary/Supplementary Figure 1 (3).TIF]

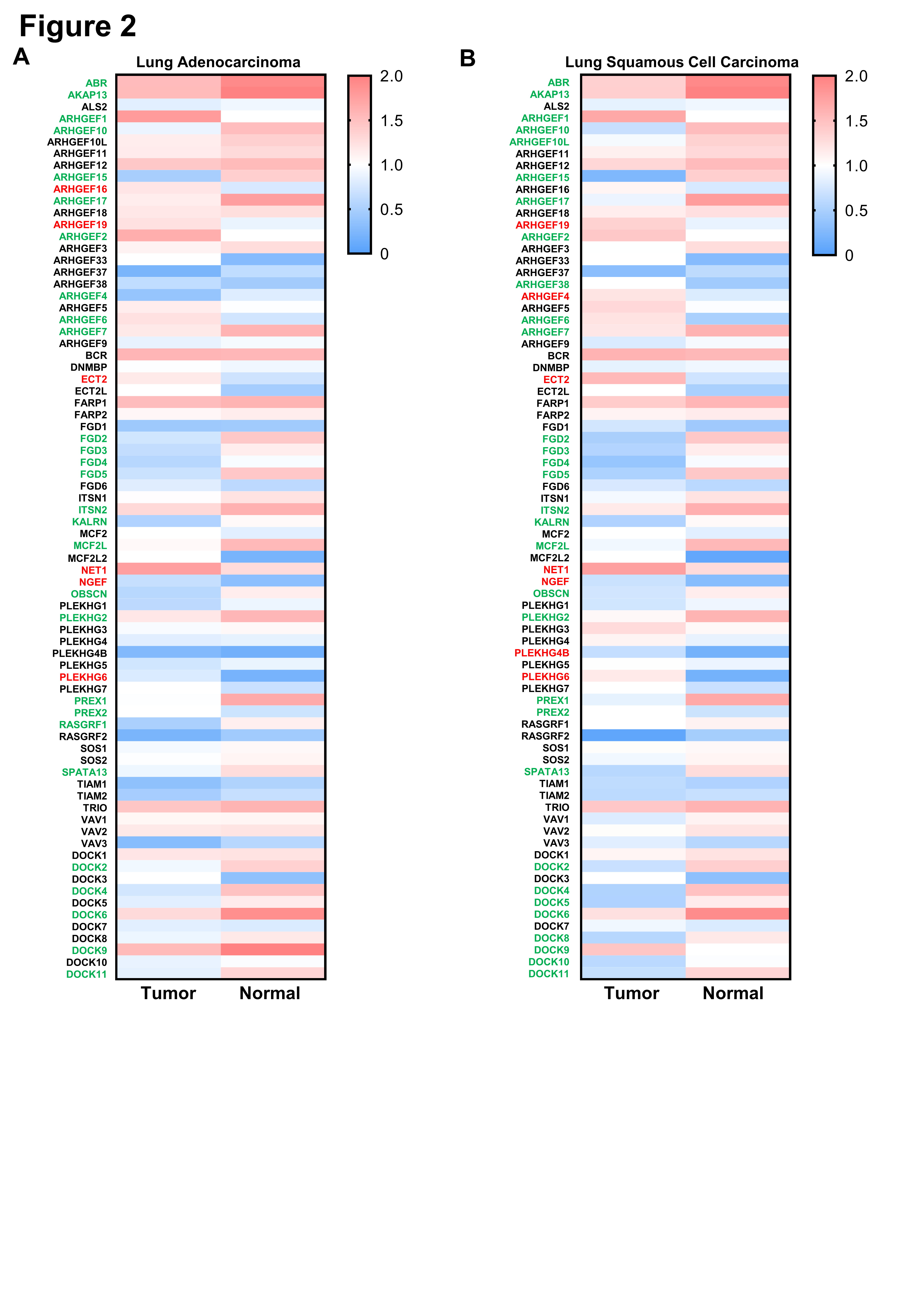

Supplement: Supplemental Material [file KBIE_A_2006519_SM0761.zip › supplementary/Supplementary Figure 2 (1).TIF]

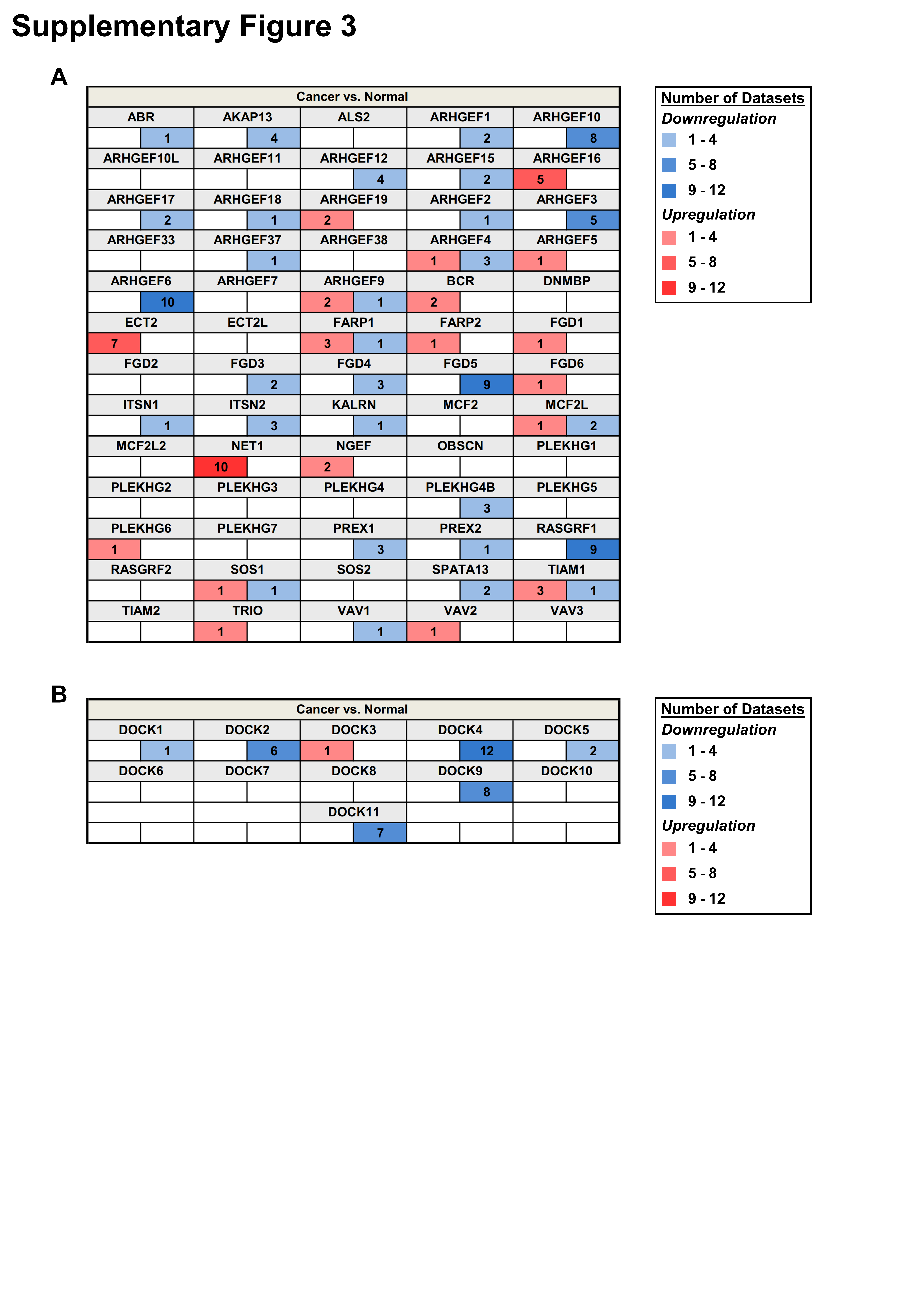

Supplement: Supplemental Material [file KBIE_A_2006519_SM0761.zip › supplementary/Supplementary Figure 3.TIF]

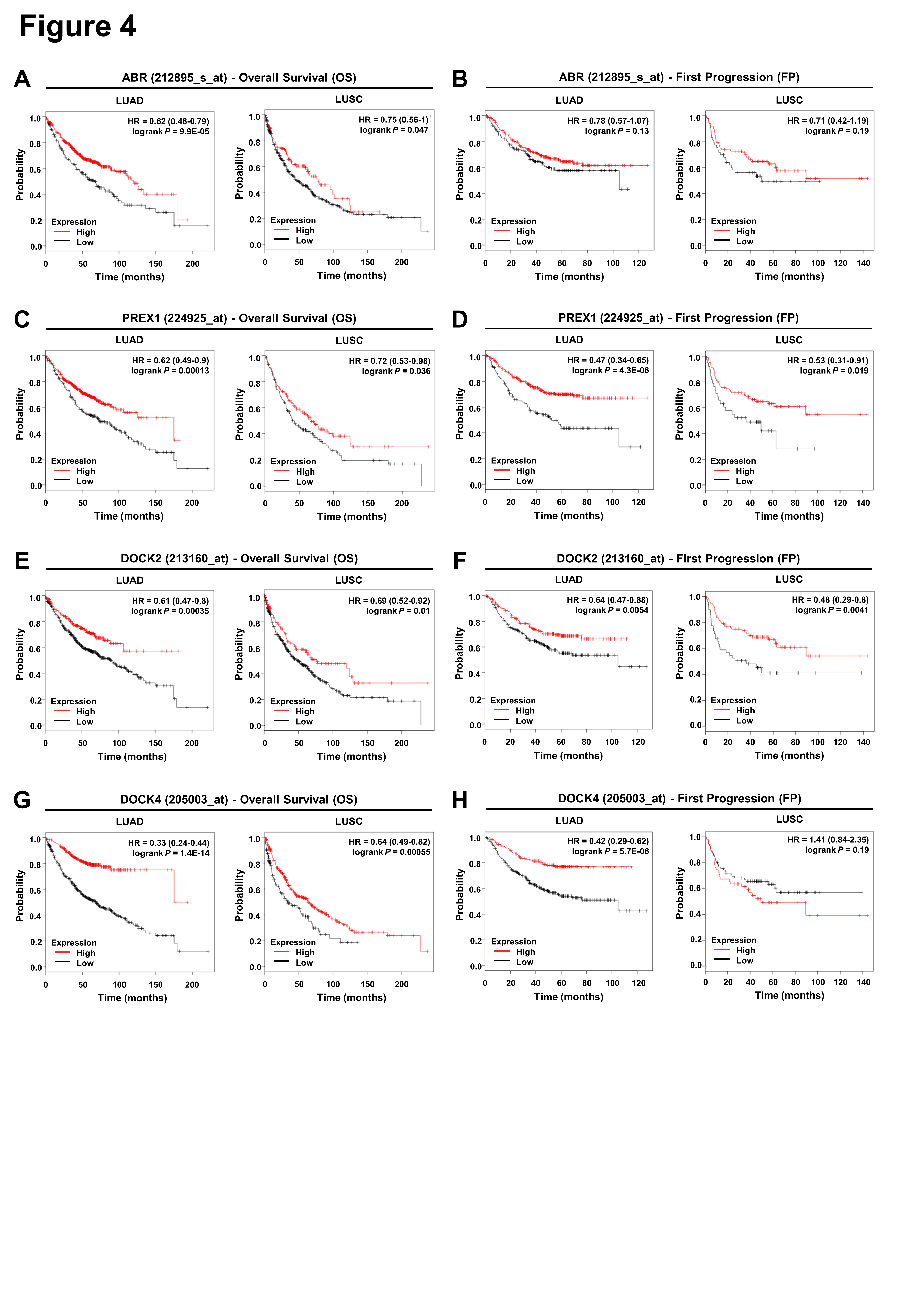

Supplement: Supplemental Material [file KBIE_A_2006519_SM0761.zip › supplementary/Supplementary Figure 4.TIF]

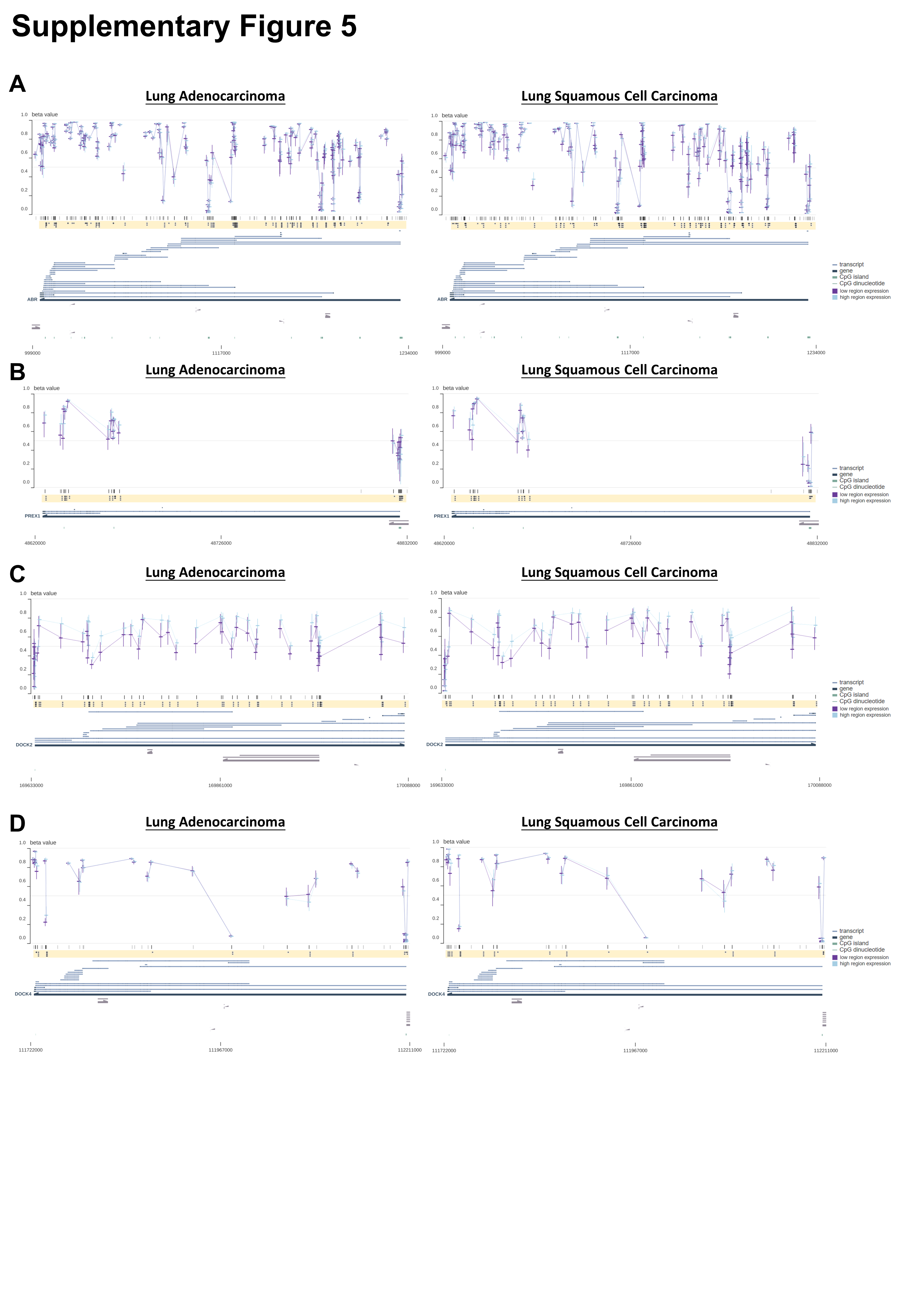

Supplement: Supplemental Material [file KBIE_A_2006519_SM0761.zip › supplementary/Supplementary_Figure_5.TIF]

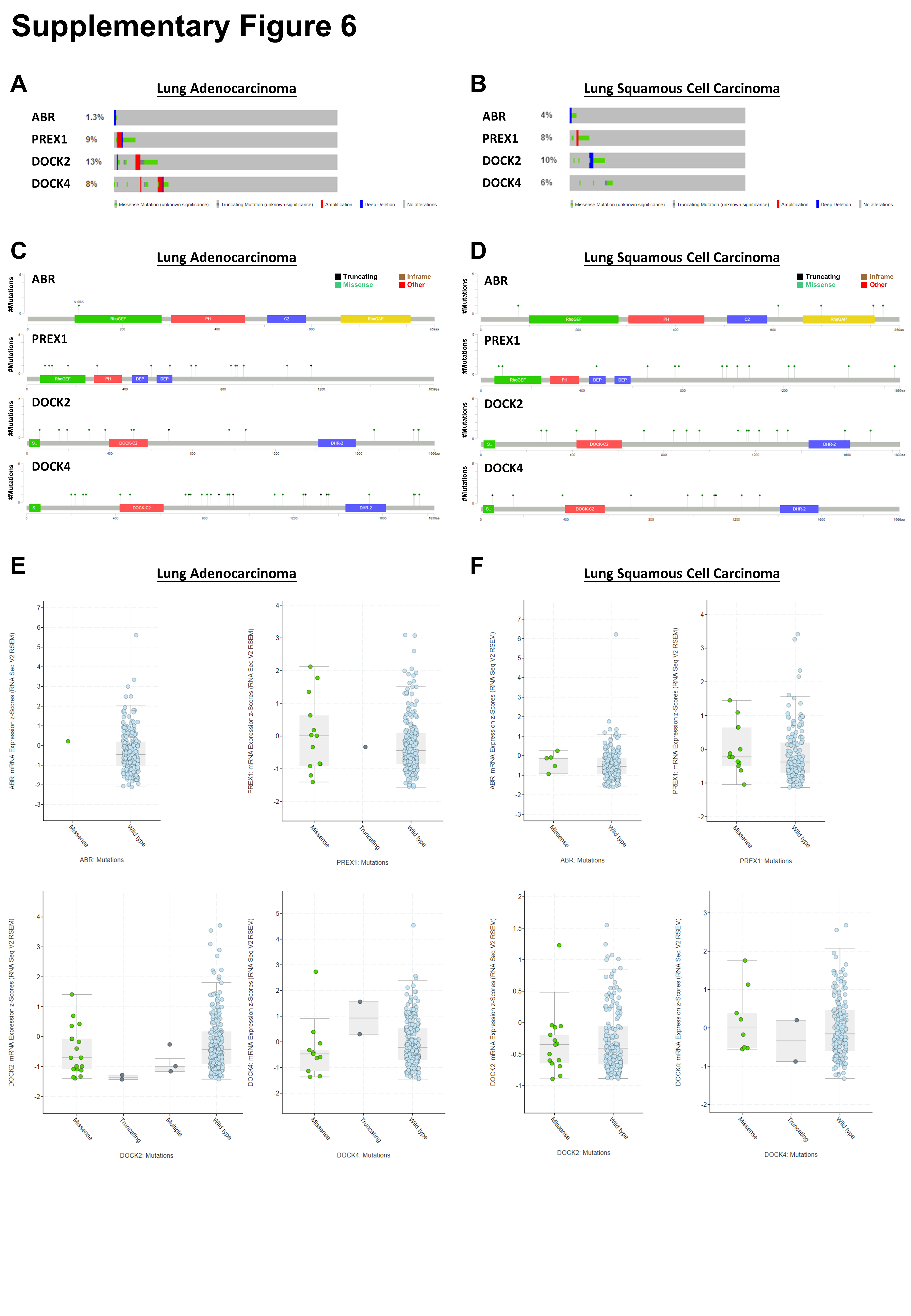

Supplement: Supplemental Material [file KBIE_A_2006519_SM0761.zip › supplementary/Supplementary_Figure_6.TIF]
